# L- and M-cone-directed Global flash multifocal electroretinogram: conceptualization and development

**DOI:** 10.1007/s10633-026-10092-z

**Published:** 2026-03-14

**Authors:** Nandini Ravi, Henry H. L. Chan

**Affiliations:** 1 Laboratory of Experimental Optometry (Neuroscience), School of Optometry, The Hong Kong Polytechnic University, 11 Yuk Choi Road, Hung Hom, Kowloon, Hong Kong, China; 2 Centre for Myopia Research, School of Optometry, The Hong Kong Polytechnic University, Hong Kong, China; 3 Colour Imaging and Metaverse Research Centre, The Hong Kong Polytechnic University, Hong Kong SAR, China; 4 Centre for Eye and Vision Research (CEVR), 17W Hong Kong Science Park, Hong Kong SAR, China

**Keywords:** L/M cone response ratio, Cone photoreceptors, Silent substitution, LED stimulator, Multifocal ERG

## Abstract

**Background and purpose:**

Multifocal Electroretinogram (mfERG) applies a fast flicker-based stimulation on the human retina and is considered a valuable tool in studying the cone photoreceptor functional pathways. At the same time, the global flash paradigm of the mfERG (MOFO mfERG) is found to be advantageous in the simultaneous evaluation of retinal responses arising from outer (direct component, DC) and inner (induced component, IC) retinal levels. Incorporating a silent substitution stimulus to the MOFO mfERG remains unexplored yet has broad potential utility. The present study aimed to develop an L- and M-cone directed global flash mfERG.

**Methods:**

A silent substitution stimulus was created appropriate for the commercially available LED monitor. Using the conventional 96% contrast MOFO mfERG as a reference, L- and M-cone directed MOFO mfERG with 19-hexagon stimulation was created and tested in 36 Chinese adults with normal colour vision. Experimental validation was conducted using high-intensity red and green light adaptation to simulate colour vision deficiency.

**Results:**

Mathematical validation was performed by calculating and ensuring that proper cone quantal catches are achieved at both targeted and non-targeted cone photoreceptors. The cone response amplitude from participants with simulated protanopia and deuteranopia showed a reduction of up to 50% (p < 0.05) following pigment bleach due to light adaptation. The mean L/M cone amplitude ratio for the Chinese adults concerning the DC and IC was 0.84 ± 0.29 and 0.77 ± 0.32, respectively, for all rings combined. The M-cone amplitudes were higher than that of the L-cone. The M-cone responses were phase-advanced or faster compared to the L-cone responses (p < 0.001).

**Conclusion:**

The L- and M-cone-directed global flash mfERG protocols may provide valuable details on the specific cone-related outer and inner retinal responses and hold extensive utility in cone-related diseases and the evaluation of colour vision.

## Introduction

The multifocal electroretinogram (mfERG), developed by Sutter and Tran, utilizes a method of binary pseudo-random visual stimulation in which the retinal regions are stimulated independently, and the local responses are mathematically extracted using a cross-correlation technique [1]. Such fast flicker-based stimulation on the human retina is found to be extensively useful in comprehending the linear and non-linear retinal responses [2]. The mfERG software is programmed to provide first- and second-order kernel responses. Previous studies have highlighted the usefulness of first-order kernel responses in understanding photoreceptor-related changes [3] and the second-order kernel responses in understanding retinal diseases affecting the inner retina [4, 5]. The poor signal-to-noise ratio and complexity of the second-order kernel responses impose difficulty on clinicians and researchers in effectively analyzing the non-linear retinal responses. One solution to this problem is the development of the Global Flash Multifocal Electroretinogram (MOFO mfERG), which helps in studying the non-linearities arising from the inner retina [6, 7]. The MOFO mfERG works by incorporating dark and global flash frames into the standard m-frame of the mfERG, thereby evaluating the fast adaptive mechanisms. The effects of luminance combinations of the video frames to achieve optimal (direct component (DC) and induced component (IC)) characteristics are studied for an achromatic stimulus [8]. Likewise, retinal responses to coloured stimuli have various implications in terms of studying the specific cone-specific and post-receptoral pathways [2, 9]. The ISCEV extended protocol to test the S-cones, uses a bright background to saturate the rods, L-, M- cones and a dim stimulus at short wavelength eliciting S-cone response [10].

Matching the number of photoisomerization using a specific stimulus to control excitation in specific photoreceptors is called the silent substitution [11, 12]. The modulation of the long or middle wavelength sensitive cones at identical cone contrast using this method in multifocal stimulus was studied previously [13–18]. The L- and M-cone-specific mfERG amplitudes and their relations to retinal topography were studied and the cone-specific amplitude ratios were found to be dependent on the retinal eccentricity [13–15]. Since the mfERG is predominantly shaped by the photoreceptors (N1) and ON bipolar cells (P1) [5], the L/M cone amplitude ratios were speculated to correspond to the number of cones in the retina [9]. Physically, the number of L-cones dominates the number of M-cones in the peripheral retina which could be the explanation for variations in colour discrimination tasks in central versus peripheral retina [19, 20]. This anatomical property is usually reflected in electrophysiological studies with a higher mean peripheral L/M cone ratio (2.3 ± 2.0) than that in the central region (1.4 ± 0.6) [13]. The mfERG waveform mainly reflects the functional integrity of the outer retina. However, inner retinal contributions also influence the shape and amplitude of mfERG [21]. The way in which cone-specific ERGs are processed at the level of outer and inner retina might be convoluted, distinctive and remain unexplored. Global flash (MOFO) mfERG technique using a specific sequence of stimulus frames allow the ERG signals to be separated into components arising predominantly from the distal retina and those originating from the inner retina [6, 7]. Silent substitution enables precise control over the spectral composition of light stimuli, making it easier to analyze individual photoreceptor responses [12]. In this study, an attempt was made to combine a silent substitution stimulus into the global flash stimulation technique for the development of a specific MOFO mfERG paradigm as an approach to distinctly study cone-directed outer and inner retinal contributions. This combination allows for a detailed examination of the temporal interactions and complexities involved in inner retinal processing. The current cone-directed multifocal ERG has been developed to elucidate retinal responses across various topographical regions of the retina. The objectives of this study are:

i. To design the cone-specific stimulus paradigm using the silent substitution technique
ii. To validate the developed protocol theoretically and experimentally
iii. To obtain the L/M cone response ratios using the developed technique.

## Methodology

### Computation of cone-directed silent substitution stimulus

**Fig. 1:**
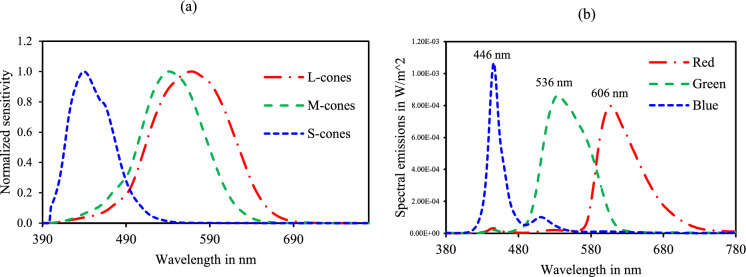
**a** 10-degree Stockman and Sharpe cone fundamentals [22]. **b** R/G/B spectral emissions from the monitor

The normalized Stockman and Sharpe (Fig. 1a) psychophysically obtained colour matching functions for the central 10 degrees were used as the cone fundamentals [22]. The emission spectrum (Fig. 1b) for the three primaries from the LED monitor was obtained using the Topcon Spectroradiometer (Model SR-3AR, Topcon Technohouse Corporation, Tokyo, Japan). The measurements of the spectral emission ranged from 380 to 780 nm with a wavelength resolution of 1 nm at a measuring angle of 2 degrees. The measurement angle with respect to the luminance ranges was selected based on the guidelines provided by the manufacturer. [23]. The energy units of the spectral radiance were obtained in W/sr/m^2^/nm. The spectroradiometer was placed at a distance of 40 cm, the same as that of the viewing distance of the participants during mfERG recordings. The energy units were converted into the number of quanta using Planck’s equation. The silent substitution stimulus was created by integrating the cone fundamentals with the V(λ) incorporated emission spectrum and setting specific conditions to the general matrix Eq. (1) which is based on the principle of univariance which is described elsewhere [24]. The detailed computation, which is given in Appendix 1, is based on the coefficient matrix.

1 $$\left( {\begin{array}{*{20}c} {\Delta r} \\ {\Delta g} \\ {\Delta b} \\ \end{array} } \right) = rgb\_LMS \times\left( {\begin{array}{*{20}c} {\Delta L} \\ {\Delta M} \\ {\Delta S} \\ \end{array} } \right)$$

where $\Delta r$, $\Delta g$, $\Delta b$ are the changes in intensities of red, green, and blue primaries, respectively and $\Delta L, \Delta M, \Delta S$ are the changes in the quantal catches in the L-, M- and S-cone, respectively. By giving specific conditions to Eq. 1 such as the change in L-cone to be set at maximum (where $\Delta \mathrm{L}=$ $\Delta \mathrm{Lmax},$ and a change in other cones $\Delta M$=0 and $\Delta S$ =0) to obtain cone directed conditions. Due to the working range of red LED cannot be set beyond the level of green LED, in Eqs. (2) and (3), the change in red LED is set as maximum, i.e., the $\Delta r$ is set as 1 for both the L- and M-cone-directed conditions.

2 $$\left( {\begin{array}{*{20}c} {1.0} \\ {\Delta g} \\ {\Delta b} \\ \end{array} } \right) = rgb\_LMS \times \left( {\begin{array}{*{20}c} {\Delta Lmax} \\ 0 \\ 0 \\ \end{array} } \right){-\!\!-}Condition \, for \, L - cone \, isolation$$

3 $$\left( {\begin{array}{*{20}c} {1.0} \\ {\Delta g} \\ {\Delta b} \\ \end{array} } \right) = rgb\_LMS \times \left( {\begin{array}{*{20}c} 0 \\ {\Delta M\max } \\ 0 \\ \end{array} } \right){-\!\!-}Condition \, for \, M - cone \, isolation$$

Solving Eqs. 2, 3 the values of $\Delta \mathrm{Lmax}$, $\Delta \mathrm{g}$ and $\Delta \mathrm{b}$ are obtained. Each state of the mfERG is a combination of red, green and blue primaries. The values of $\Delta \mathrm{r}$, $\Delta \mathrm{g}$ and $\Delta \mathrm{b}$ are percentage changes in red, green, and blue primaries, which are used to set the 2-flicker states of the multifocal frame for the corresponding cone-directed condition. The strength of the cone-directed stimulus is usually denoted by its respective cone contrast, which is given by the formula,

$$\text{Cone Contrast }= \frac{\text{excitation maximum }-\text{ excitation minimum}}{\text{excitation maximum }+\text{ excitation minimum}} \times 100\%$$

where the maximum and minimum excitations for the L- and M-cone-directed conditions were computed using the coefficient matrix used in the calculations. For the gamut of the LED monitor used in the present study, the cone contrasts achievable for the L- and M-cone-directed conditions were 34.65% and 34.8% respectively. The luminance contrasts of the cone-directed stimuli are expressed in terms of Michelson’s contrast. The luminance contrast of the L-cone directed stimulus condition was 16.7% and the same for the M-cone-directed stimulus condition was 17.3%.

### Suitability of the commercially available LED monitor for silent substitution research and system calibrations

**Fig. 2:**
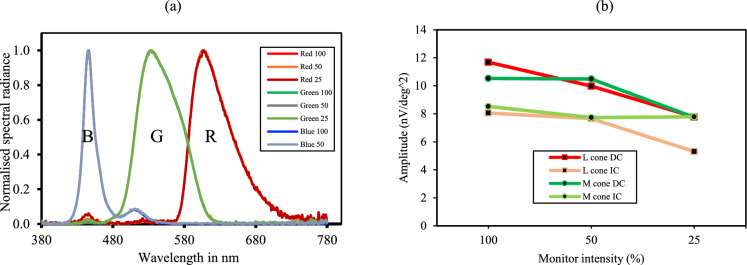
**a** Showing spectral homogeneity of the LEDs. **b** Showing the monitor intensity response series

The success of the silent substitution method implied in the study depends on the calibration of the colour LED monitor, by establishing a relationship between the measured output luminance and the digital input in the mfERG system software. The main reason for such discrepancies originates from the various configurations from different studies assigned to the stimulus, such as the spatial, temporal, and chromatic properties [25]. For the mfERG recording used in this study, a Samsung S24E650PL with a monitor resolution of 1280 × 720 pixels was used. The scale in the mfERG software for the red, green, and blue LED panel had a different luminance range. Red LED had a luminance range from 2 to 54 cd/m^2^, green LED had a luminance range of 3 to 185 cd/m^2^, and blue LED had a luminance range of 3 to 22 cd/m^2^. Since the mentioned digital inputs might differ from the actual luminance output achieved, the Konica Minolta handheld Spot Photometer (LS-110) was used to check the luminance achieved at 40 cm. The LED panel of the monitor was divided into 4 equal quadrants and four random points within the quadrants were used as a target to check the luminance for red, green, and blue emissions. A conversion table was created with 1 cd/m^2^ step which served as a guide to set the required time-averaged luminance for the individual cone-directed stimulus conditions. The colour reproducibility across various light levels were measured to check the spectral homogeneity [26]. No major drift was noticed between 100%, 50% and 25% of RGB primary spectral power distributions (Fig. 2a). This confirmed the ability and suitability of the monitor for applying the silent substitution paradigm while leaving non-targeted photoreceptors unexcited.

To validate the relative amplitude of the L- and M-cone MOFO mfERG with changes in the luminance level, a monitor intensity response trial was performed at three brightness levels which were 100%, 50% and 25%. The DC amplitude of L- and M-cone amplitude decreased in a similar fashion but for the IC component, only the L-cone amplitude dropped drastically compared with the M-cone (Fig. 2b). Considering this reduction in L-cone IC amplitude at 25% brightness level, the monitor brightness for this condition was kept at the level of 100% for all the ERG recordings.

### Order of video frames in cone-directed global flash multifocal ERG paradigm

**Fig. 3:**
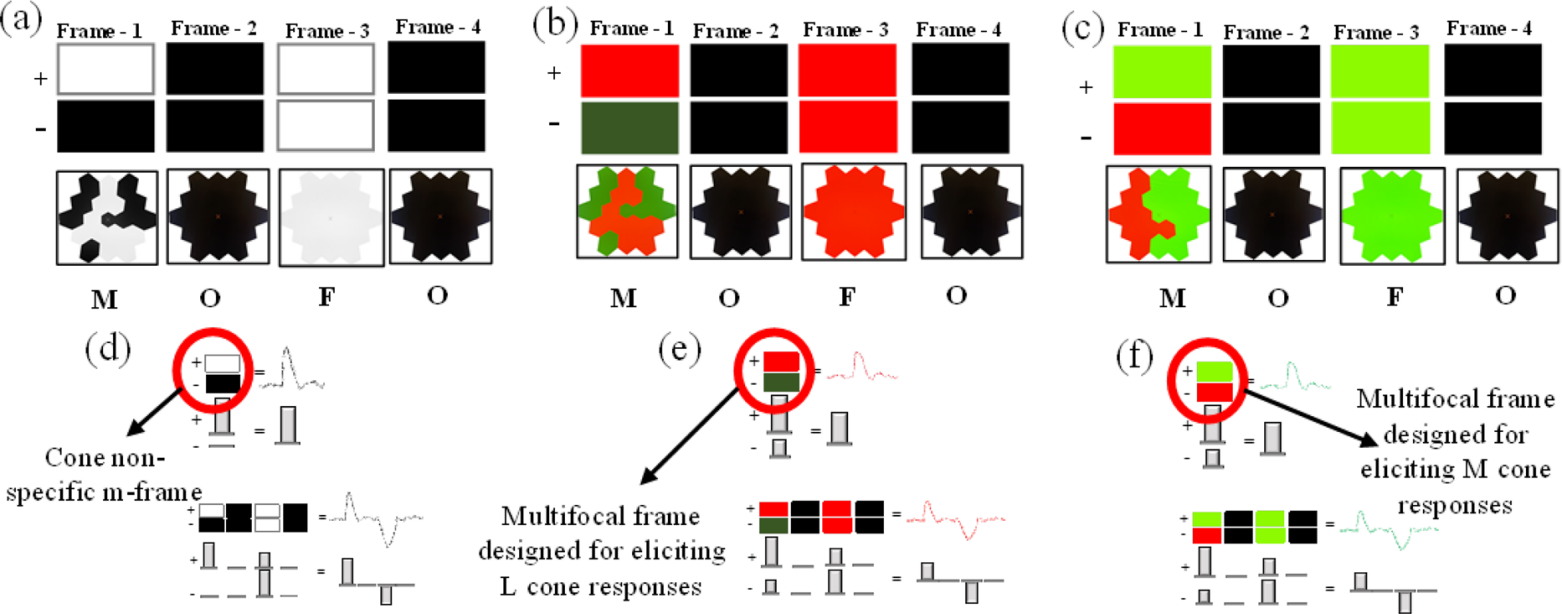
Four video frames of: **a** Black and white global flash mfERG, **b** L-cone-specific global flash mfERG, **c** M-cone-specific global flash mfERG; Demonstration of DC and IC characteristics of **d** Black and white MOFO mfERG, **e** L-cone-directed MOFO mfERG, and **f** M-cone-directed MOFO mfERG

The global flash (MOFO) mfERG paradigm consisted of a 19-hexagon pattern that incorporated four video frames in each m-sequence step. A comparison of a 61- vs. a 19-hexagon trial was performed before the start of the study. As a good SNR is important in this measurement, the 19-hexagon stimulus was applied although the resolution remained compromised to a limited extent. A single frame of the mfERG contains two states denoted as 1-plus and 0-minus. The first frame is a focal frame which repeats pseudo-randomly followed by a dark frame. The third frame is assigned such that all the hexagons are uniformly or globally flashed which is then followed by another dark frame. It is essential to note that only the first frame of the global flash mfERG paradigm follows a binary m- sequence technique (Fig. 3). The plus state of the multifocal frame was always kept as the brightest to avoid changes in the polarity of the waveforms. The arrangements of dark frames distinguished and elicited two responses, the DC and IC. The DC is the local response to the focal flashes which is analogous to the standard multifocal ERGs. The IC is the response elicited by the global flash with and without the effect of focal flashes [27]. The acronym “MOFO” is used to represent the paradigm which refers to the multifocal (M) frame, followed by an OFF (O) dark frame, a global flash (F) frame, and concluding with another OFF (O) dark frame.

The global flash frame arrangements described by Sutter et al. [7], served as a reference for the frame arrangements of the cone-directed global flash MOFO mfERG paradigm (Fig. 3a, b and c). The first frame of the L-cone condition is the multifocal frame, which has the red and green fast flicker-based binary pseudo-random visual stimulation, whose luminance is computed by the silent substitution paradigm as explained in Sect. "Computation of cone-directed silent substitution stimulus". The monitor remained black during the dark frame with a luminance less than 2 cd/m^2^ for the second and fourth frames. The global flash frame of the L-cone condition had all the hexagons take a completely red state with a luminance of 27. 5 cd/m^2^. (Fig. 3b). For the M-cone condition, the first frame had the green and red alternating fast flicker stimulation, whose luminance is computed by the silent substitution paradigm. The second and fourth frames also remained dark. All the hexagons in the third frame took the green state with a luminance of 78.9 cd/m^2^ (Fig. 3c). The background luminance, beyond the stimulus zone had a luminance of 80 cd/m^2^ in both the conditions. Because the m-frame uses silent substitution to adapt only the L-cones or M-cones, and the Global Flash uses a matched chromatic spectrum (red for L-cone-directed stimuli and green for M-cone-directed stimuli) to preferentially probe the respective cones, the resulting interaction term (IC) is physiologically constrained to reflect the adaptive properties of the L-cone pathway and its associated inner-retinal circuitry. (Fig. 3d, e and f).

### Study participants for mfERG recording

The mfERG conditions were tested in 36 Chinese adult participants. The mean age of the participants was 20.43± 2.1 years. All the participants had a best corrected visual acuity of 1.0 (LogMAR) or better and the mean non-cycloplegic refraction at the corneal plane was -3.08 ± 2.64 D. The participants had normal ocular health and colour vision was tested by Ishihara and Oculus HMC anomaloscope. This study was approved by The Hong Kong Polytechnic University Institutional Review Board (Reference number: HSEARS20240205003) and all procedures followed the tenets of the Declaration of Helsinki. Written consents were collected from the subjects. The pupils were dilated using 1% tropicamide (Mydriacyl, Alcon Eye Care UK Ltd.) to achieve at least 7 mm pupil diameter. DTL electrodes placed at the lower limbus of the eye acted as the active and gold cup skin electrodes placed at the forehead and ipsilateral temple served as the ground and reference electrodes respectively. The skin preparation and electrode placements were done according to ISCEV standards. The VERIS software (6.0.6d19; EDI, San Mateo, CA, USA) was used for mfERG recordings. The m-sequence of 2^11^–1 (recording time: 2 min 17s) protocol with 19 scaled hexagonal elements was used to notice acceptable waveforms. The vertical and horizontal dimensions of the monitor were 29 × 52 cm. The mfERG stimulus dimensions were 31.5 × 29 cm enclosing a visual angle of 39.85. The signals were amplified 100,000 times, band pass filtered from 10 to 300 Hz and digitized at a sampling rate of 1000 Hz. All participants stayed in an ambient well-lit room (300 lx) for at least 10 min before the start of ERG recording, and the background stimulus luminance (80 cd/m^2^) was chosen to eliminate rod intrusions. A fixation cross was set in the central hexagon for gaze stabilization and to facilitate the ability of the subject to maintain fixation, the stimulation was broken up into 16 segments each lasting 9.0s.

### Theoretical validation

The multifocal frame of the mfERG involves flicker between two stimuli (State 1 and State 2). To verify whether the silent substitution condition is met, the following conditions must be satisfied.

1. In the targeted cone, there has to be a considerable difference in the cone quantal catch achieved between State 1 and State 2.
2. In the non-targeted cones, the difference in the quantal catch achieved between State 1 and State 2 has to be zero; thus, essentially ensuring the silence of the non-targeted cones.

For example, in the L-cone-directed condition, there should be a considerable difference in the L cone (targeted cone) quantal catches between State 1 and State 2 while the difference in the M- and S-cones (non-targeted cones) should be zero. The mathematical calculation proving the above inequality is explained below. The cone quantal catch is the product of the change in intensity of the primary and the effective cone quantal catch of a particular cone type for the corresponding primary. The calculated ∆r, ∆g, and ∆b values were 1.0, 0.25, and 0.003, respectively, and were used to set the respective luminance for the L-cone-directed stimulus condition.

The L-cone quantal catches for the L-cone-directed condition when flickering between State 1 and State 2 were obtained as follows:

**Table:** 

State 1 (RED)	Red primary: ON Green and Blue: OFF	∆r × LR = 1.0 × 180,561	=	180,561	quanta/ L-cone/ s
State 2 (GREEN)	Red primary: OFF Green and	∆g × LG = 0.25 × 274,969	=	68,105	quanta/ L-cone/ s
State 2 (BLUE)	Blue primary: ON	∆b × LB = 0.003 × 18,698	=	72	quanta/ L-cone/ s

The difference in the L-cone quantal catch achieved between State 1 (red primary = 180,561) and State 2 (green and blue primaries = 68,105 + 72 = 112,384) quanta. To verify the achievement of silent substitution, the difference in the M-and S-cone quantal catches for L-cone-directed stimulus conditions was also calculated and compared.

The M-cone quantal catches for the L-cone-directed stimulus condition were obtained as follows:

**Table:** 

State 1 (RED)	Red primary: ON Green and Blue: OFF	∆r × MR = 1.0 × 59,702	=	59,702	quanta/ M-cone/ s
State 2 (GREEN)	Red primary: OFF	∆g × MG = 0.25 × 240,706	=	59,619	quanta/ M-cone/ s
State 2 (BLUE)	Green and Blue primary: ON	∆b × MB = 0.003 × 21,293	=	82	quanta/ M-cone/ s

The difference in the M-cone quantal catch achieved between State 1 (red primary = 59,702) and State 2 (green and blue primaries = 59,619 + 82 = 0) quanta.

The S-cone quantal catches for the L-cone-directed stimulus condition were obtained as follows:

**Table:** 

State 1 (RED)	Red primary: ON Green and Blue: OFF	∆r × SR = 1.0 × 471	=	471	quanta/ S-cone/ s
State 2 (GREEN)	Red primary: OFF Green and	∆g × SG = 0.25 × 1685	=	417	quanta/ S-cone/ s
State 2 (BLUE)	Blue primary: ON	∆b × SB = 0.003 × 13,844	=	53	quanta/ S-cone/ s

The difference in the S-cone quantal catch achieved between State 1 (red primary = 471) and State 2 (green and blue primaries = 417 + 53 = 0) quanta. From the above equations when the red (State 1) and green (State 2) lights are altered, the change in quantal catches is nearly zero for the M- and S-cone, while it is maximum for L-cone. Thus L-cone-directed stimulus condition is theoretically verified. Similarly, the M-cone-directed stimulus condition was also theoretically validated. The splatter contrast on the non-targeted cones was also computed using spectroradiometric measurements of the ON and OFF states for both the L and M cone-directed stimulus conditions. As seen in Table 1, the targeted L and M photoreceptors had a contrast of 41.85% and 43.95% respectively, while the splatter contrast in non-targeted cones was less than 5% in general.

**Table 1:** Showing cone contrast in targeted versus non-targeted cone photoreceptors

Splatter	L cone	M cone	S cone
L cone condition	41.85%	−4.71%	3%
M cone condition	−2.51%	43.95%	−2.48%

The developed silent substitution paradigm was validated theoretically and experimentally, and the DC- and IC-based L/M cone amplitude ratios were reported for the corresponding subjects in the following section.

## Results

### Experimental validation

**Fig. 4:**
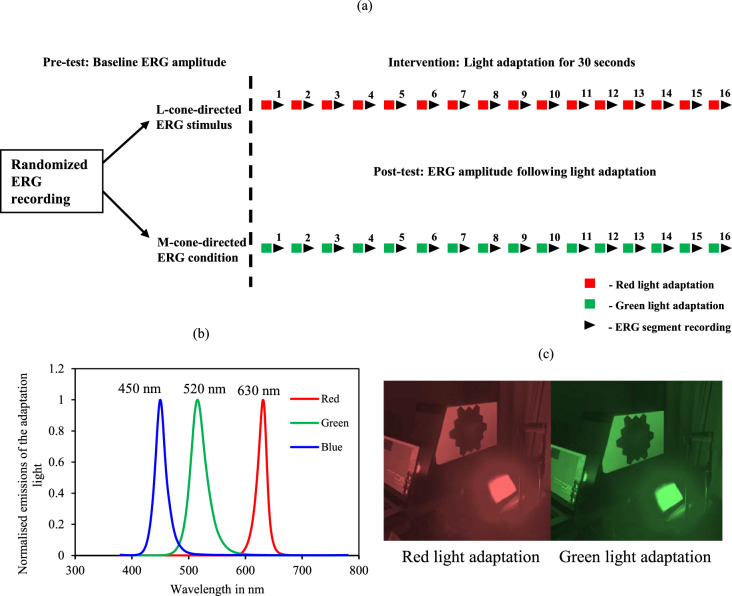
**a** Schematic representation of the light adaptation experiment. **b** The emission spectrum of the light used for the adaptation experiment. **c** Experimental setup of red and green light adaptation

Cone-directed stimulus are conventionally validated in dichromats where there is a signal obtained below noise level for such stimulus. As the prevalence of dichromats is relatively low, which is one in 30,000 in the Hong Kong Chinese population, testing the L- and M-cone-directed stimulus protocols in individuals with Protanopia (absent L-cones) and Deuteranopia (absent M cones), respectively, for validation remained impracticable. To counter this issue, light adaptation experiments were performed to temporarily simulate colour vision deficiency. To saturate the L-cones, 30s of red light (630 nm) with a luminance of 2248.5 cd/m^2^ adaptation was administered before each segment of mfERG recordings. This state of temporary loss of sensitivity to specific colour vision due to bright light adaptation was referred to as the ‘artificial protanopia [28, 29] and ‘artificial tritanopia’ which mimics partial colour vision weakness. Although, previous light adaptation conditions were not described analogous to deuteranopia, a 30s adaptation to green light (520 nm) with a luminance of 6429 cd/m^2^ to saturate the M-cones was also performed. The difference in luminance between red and green light was intended to match the radiant exposure at the retinal level. The L- and M-cone-directed ERG protocols were recorded before and after light adaptation and the amplitudes were compared. The schematic representation of light adaptation experiment is in Fig. 4a, and the spectral emissions of the commercially available LED light and the experimental setup are given in Fig. 4b and 4c. Apart from the red and green LED emissions, the blue LED emissions are shown in Fig. 4b, as it serves an essential role in generating the white stimulus used for this experiment, in combination with other LEDs. Using the cone fundamentals and the LED emissions of the adaptation light, the amount of bleach in the targeted and non-targeted photoreceptors was calculated in terms of percentage. The red LED used is computed to bleach around 84% of L-cones, 16% of M-cones and 0% S-cones. While, the green LED used bleached around 45% of L-cones, 53% of M-cones and 1% S-cones. Using the HMC anomaloscope, the adaptation-induced change in colour perception was tested (three repeated measures) in one normal colour vision observer, and the change persisted for more than 20 s (approximately 20 ± 3 s), indicating a sustained alteration of receptor and post-receptoral processing. This duration of perceptual shift supported the conclusion that a 30-s light-adaptation protocol should be sufficient to induce measurable changes in both DC- and IC-based ERG amplitudes following light adaptation. The radiant exposures of the bright stimuli remained well within established ocular radiation safety limits.

**Fig. 5:**
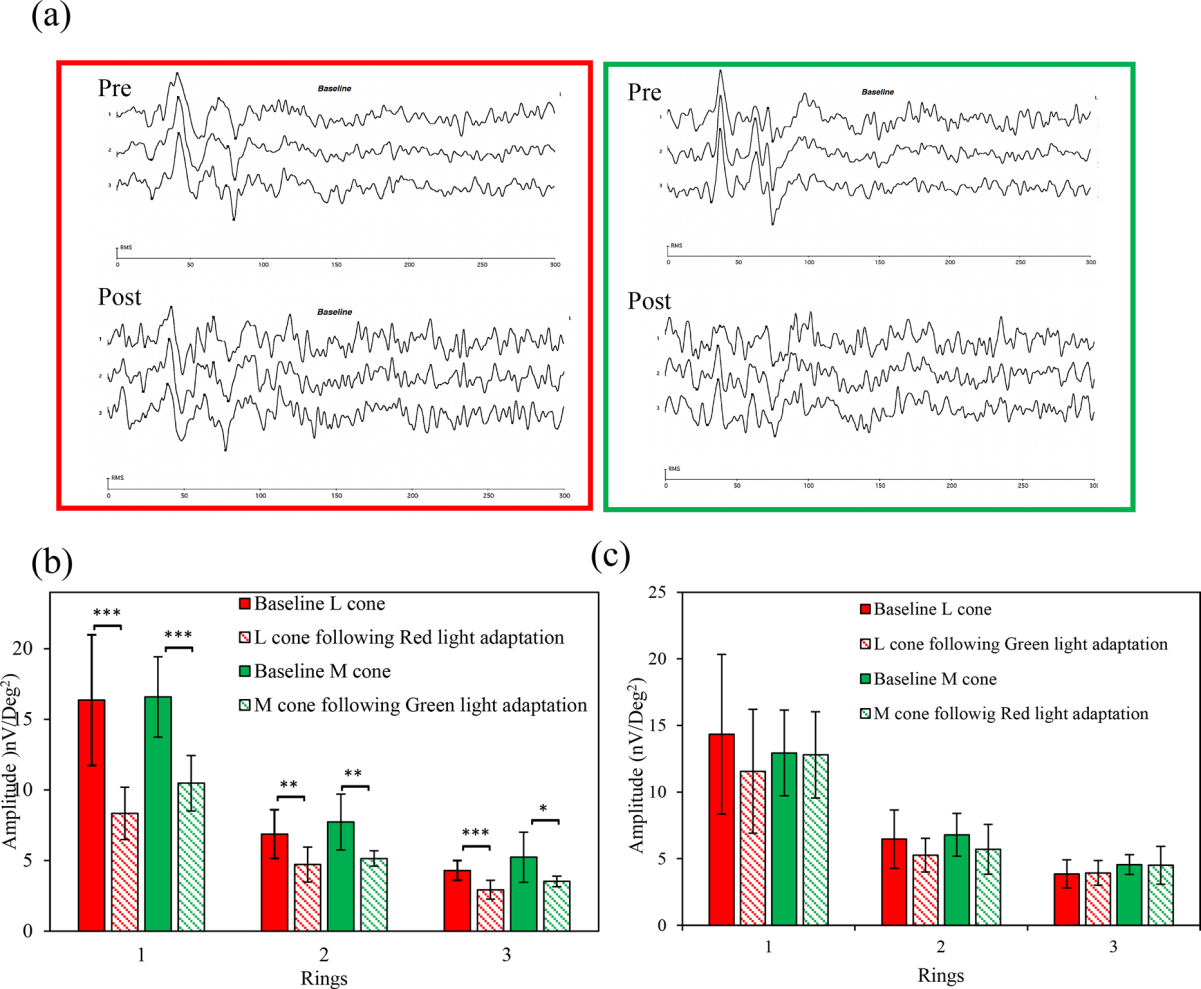
**a** A typical L- (denoted in red box) and M-cone (denoted in green box) -directed ERG waveform in pre- and post-light adaptation. **b** Comparison of ring-wise DC response amplitudes of baseline L- and M- versus post-light adaptation of respective cones. **c** Comparison of ring-wise DC response amplitudes of baseline non-targeted cones versus post-light adaptation for non-targeted cones

The baseline L- and M-cone-directed ERG amplitudes were compared to the post-light adaptation amplitudes for the ring 1 (central 0°-10°), ring 2 (mid-peripheral 10°-24°), and ring 3 (peripheral 24°-40° rings) in 8 eyes from subjects randomly recruited from the 36 participants. The pre- and post-light-adapted ERG response amplitude data for all the conditions were normally distributed, hence parametric statistical, pair-wise comparisons were applied. A sample waveform before and after light adaptation for L- and M-cone-directed stimulus conditions is shown in Fig. 5A. It was noted that, following a brief light adaptation before each mfERG recording segment, the signal-to-noise ratio (SNR) decreased significantly. A statistically significant reduction for the DC in L- and M-cone amplitude (paired t-test p < 0.001) was noted following red and green light adaptation, respectively (Fig. 5B and 5C). Although there was a generalised reduction in the response amplitudes from baseline to post-adaptation in the IC, it reached a statistically significant difference only in the ring 1 and ring 2 (p < 0.05) for L-cone, and only in the ring 2 (p < 0.05) of the M-cone. In contrast, the DC responses of non-targeted cones from ring 1 to 3 were not significantly different between pre- and post-red or green light adaptation (all p < 0.05). Additionally, to ensure that the amplitude reduction observed was specific to colour-deficiency simulation and not due to light adaptation, a control experiment was performed in which luminance-matched white light adaptation was administered, and both L- and M-cone responses were elicited. Following 30 min of white light adaptation, no selective amplitude reductions were observed compared to the baseline amplitudes. The change in amplitudes in the non-targeted cone types, i.e., L-cone amplitude following green light adaptation and M-cone amplitude following red light adaptation, was found to be statistically insignificant (paired t-test) as seen in Fig. 5a and 5b.

### L- and M-cone MOFO mfERG responses

**Fig. 6:**
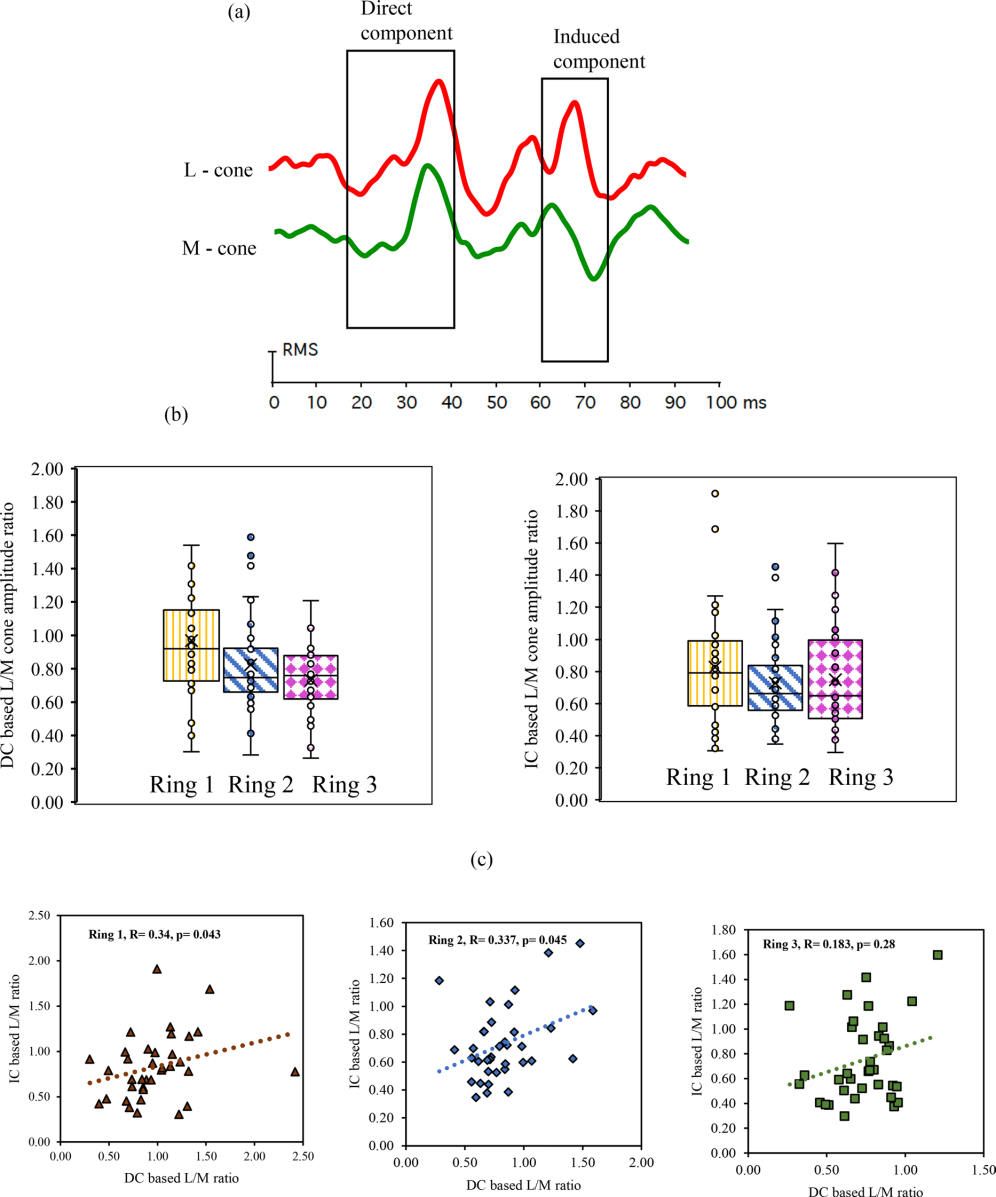
**a** Demonstration of typical L- and M-cone-based MOFO waveforms. Distribution of **b** DC-based L/M cone response ratio (pattern fill) and IC-based L/M cone response ratio (solid fill) across retinal eccentricity **c** The correlation between DC versus IC based L/M cone ratio in Ring 1, 2 and 3

The L- and M-cone-based MOFO mfERG waveform consisted of two distinct components (DC and IC), as shown in Fig. 6a. The SNR was calculated as a fraction of signal epoch to noise epoch, and only the waveforms with an SNR of 1.5 and above were chosen for L/M cone amplitude ratio analysis. The range from the initial negative wave (N1) to the positive peak (P1) was calculated as the DC, and the subsequent range from the positive peak (P2) to the negative wave (N2) was considered as the IC. The ring analysis corresponded to the different stimulus locations and the mfERG amplitudes reduced from centre to periphery. The L- and M-cone-based response amplitude data were not normally distributed. The median and inter-quartile range of the DC and IC amplitudes and latencies are given in Table 1(Table 2).

**Table 2:** Median (IQR) Amplitude and Latency data for the L- and M-cone responses

		DC amplitude (nV/deg^2^)	DC latency (ms)	IC amplitude (nV/deg^2^)	IC latency (ms)
L-cone	Ring 1	12.09 (6.10)	40.45 (3.04)	9.98 (6.71)	72.91 (4.69)
	Ring 2	6.057 (2.48)	40.97 (2.60)	3.952 (2.94)	71.87 (8.08)
	Ring 3	3.505 (1.71)	40.62 (2.60)	2.567 (1.37)	64.93 (5.0)
M-cone	Ring 1	13.41 (8.07)	36.81 (1.40)	13.44 (10.42)	69.27 (4.16)
	Ring 2	7.31 (4.40)	36.63 (1.74)	6.322 (3.88)	62.153 (8.33)
	Ring 3	4.923 (2.08)	35.76 (1.65)	3.4 (2.09)	62.67 (6.17)

The median M-cone DC and IC amplitudes were significantly higher than the L-cone amplitudes (p < 0.001, Wilcoxon signed rank test) for the peripheral rings. It was noted that the DC and IC latencies were significantly lower for M-cone compared to L-cone (p < 0.001, Wilcoxon signed rank test) for all three eccentricities. The L/M cone amplitude ratios were not normally distributed and the mean L/M cone ratio of the DC component was 0.84 ± 0.29 and the IC component was 0.77 ± 0.32 for all rings combined. Figure 6b shows the distribution of DC- and IC-based L/M cone ratios for the rings 1, 2 and 3, respectively. The Wilcoxon signed rank test showed that the DC-based L/M cone response ratio was significantly higher in ring 1 (0.97 vs 0.83 p = 0.034) and ring 2 compared to IC-based L/M cone response ratio (0.83 vs 0.73 p = 0.037). A weak correlation (Spearman’s Rho) between the DC- and IC-based cone ratios are also plotted in Fig. 6c.

To assess the influence of the different mean luminance levels employed for the L- and M-cone directing conditions, an illuminance-normalised, gain-based L/M ratio (nV/Td) by dividing ERG amplitudes by the corresponding retinal illuminance (555 Td for L-cone, 832 Td for M-cone) was additionally computed. Under this normalization, the gain-based L/M ratio was approximately 45% higher than the raw amplitude-based ratio. This auxiliary metric is reported to facilitate an explicit, retinal-illuminance–adjusted interpretation of the results, complementing the primary amplitude-based analysis.

## Discussions

**Fig. 7:**
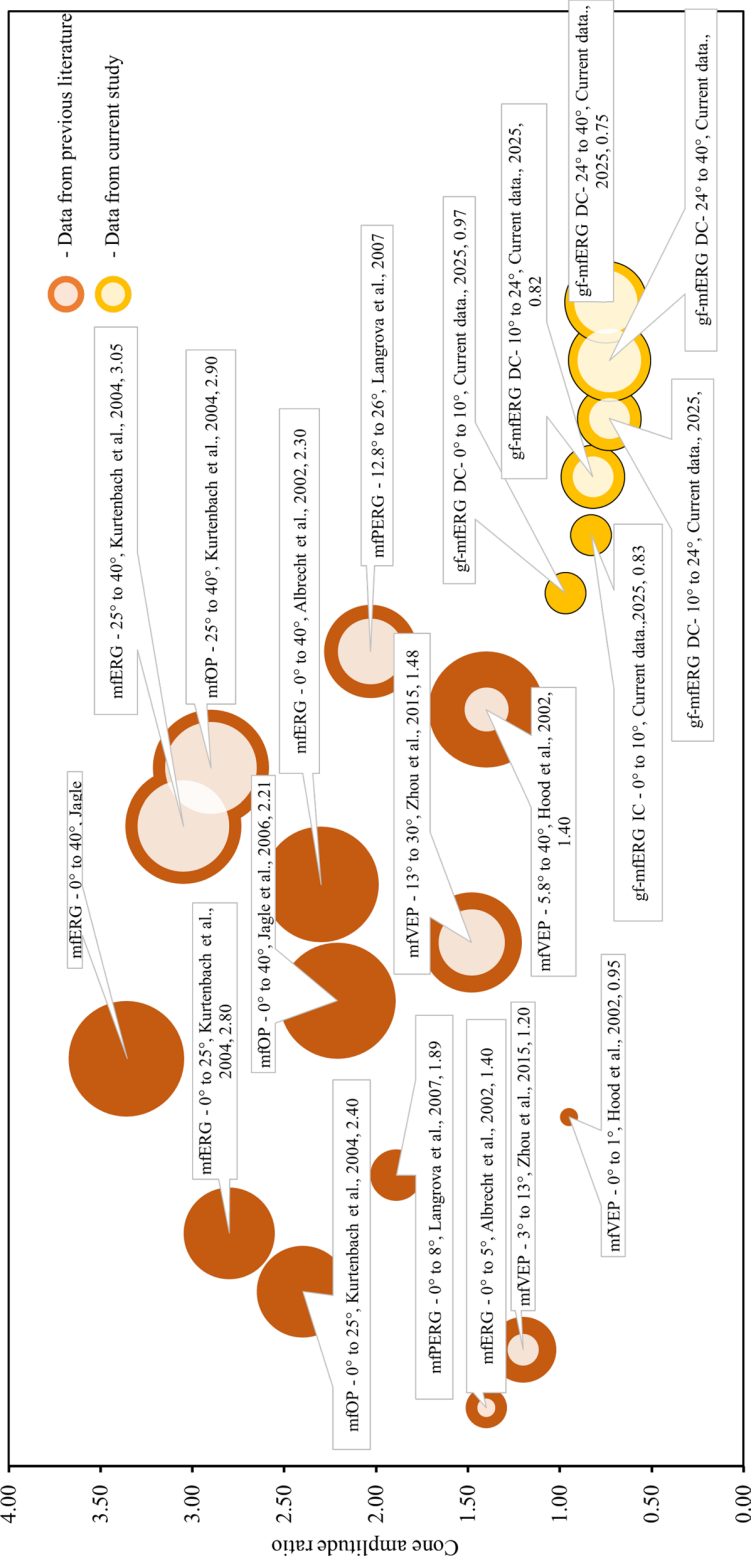
Comparison of literature showing the mean L/M cone amplitude data using various electrophysiological techniques

The multifocal electroretinogram is a valuable tool in studying the cone pathway functions [1]. The global flash mfERG paradigm brings out a distinguishing feature of the outer and inner retinal contributions [4–6]. The use of silent substitution to report the individual cone contribution is well studied in full-field flicker ERG and psychophysical techniques [9–11, 30–32]. While L- and M-cones can be viewed as a single entity due to their similar characteristics and overlapping spectra, their significant differences in retinal dystrophies, such as Best's disease and retinitis pigmentosa, should not be overlooked [33, 34]. In the present study, an attempt was made to develop a global flash mfERG paradigm with silent substitution-based stimulations for obtaining the L- and M-cone responses related to outer and inner retinal levels. The L- and M- cone contrasts obtained in this study were approximately 35% considering the gamut of the LED, this makes the L- and M-cone response amplitude data comparable. The cone-directed MOFO mfERG responses for the L- and M-cones are reported as DC and IC. The mean L/M cone response ratio was found to be lower than those reported in other studies (Fig. 7). The L- and M-cone- directed mfERG waveforms showed an acceptable repeatability, and a decent SNR was achievable within a short measurement duration in the present settings used. This may help apply these specific mfERG protocols in clinical applications. Conventional L/M ratio estimates provide a global measure but do not offer spatially resolved or layer-specific information about inner retinal function. In contrast, previous myopia studies using the MOFO paradigm has shown that: (1) reduced inner-retinal MOFO responses in the central retina can serve as a functional biomarker for future myopia onset in emmetropic children [35, 36]; (2) myopic retinal dysfunction is spatially heterogeneous, with preferential involvement of central/paracentral inner retina and relative sparing of outer retinal function in early stages [37–40]; and (3) baseline MOFO responses can predict the efficacy of myopia control interventions such as low-dose atropine [41]. By integrating silent substitution with MOFO-mfERG, the present study extends beyond a global ‘cone count’ or whole-retina L/M ratio, enabling assessment of whether L- and M-cone driven inner-retinal signals are selectively altered in specific retinal regions. Such region-specific alterations are likely to be particularly relevant for understanding cone-mediated signaling pathways that drive local eye growth in myopia.

The simulated colour vision deficiency conditions via experimental bright light adaptation provided valid evidence to demonstrate the effectiveness of this silent substitution based MOFO mfERG protocol in obtaining the targeted cone response with suppression of non-targeted cones. Bleaching the erythrolabe pigments with intense red light as a way to mimic protanopia has been studied in the past [42]. As expected, from our findings, following a temporary bleach with high intensity red and green light reduced the response amplitude to up to 50% for both the L- and M-cone-directed stimulus conditions. However, this was true only for the DC component and not for the IC component.

The IC is a mathematical derivative of how a preceding event (focal flash) alters a subsequent response (global flash). Because the focal flash uses Silent Substitution to adapt only the L-cones, and the global flash uses a matched chromatic spectrum (red for L-cone-directed stimuli and green for M-cone-directed stimuli) to preferentially probe the L-cones, the resulting interaction term (IC) is physiologically constrained to reflect the adaptive properties of the L-cone pathway and its associated inner-retinal circuitry. The unmodulated cone type acts as a 'silent bystander' and does not contribute to the difference signal that generates the kernel. Although the frame construction rationale supports the IC component being cone-directed, the light-adaptation paradigm did not produce a correspondingly robust reduction in IC amplitude. This discrepancy motivates further investigation into how light adaptation modulates the IC component. The theoretical validation in Sect. "Experimental validation" shows a major difference in the calculated quantal catches only in the targeted cones leaving the non-targeted cones silent. Since the DC is dominated by outer retinal responses related to the photoreceptors, our light adaptation experiment exhibited a change in DC of the mfERG responses. However, the IC is suggested to originate from the inner retinal layer, especially retinal ganglion cells, which received the interacted signals from both targeted and non-targeted cones. Thus, it may be why the IC cannot show significant changes under the red/green light adaptation. Also, to evaluate the changes the red and green lights bring in non-targeted cones, the M-cone DC and IC responses to high-intensity red light adaptation and L-cone DC and IC responses to high-intensity green light adaptation were measured. The results indicated that there was no notable difference in the non-targeted cones following the light adaptation. This emphasizes the effectiveness of high-intensity red and green light in simulating protanopic and deuteranopic conditions respectively.

The L/M cone ratio reported from previous studies varied considerably (Fig. 7). Different populations may be one of the factors. However, it may also be affected by the cone contrast, the ERG technique, spatial and temporal stimulus configurations, which make it difficult to draw comparisons on available L/M cone ratio data. Nevertheless, Fig. 7 shows the distribution of cone ratios across studies that used various multifocal electrophysiological techniques with the silent substitution method. The data is shown in terms of the multifocal electrophysiological technique used, the retinal region covered, and the cone ratio. The median data reported in previous literature is converted to means for ease of comparison. The mean L/M cone ratio obtained using the MOFO mfERG method in the current study is observed to be less than that of the other studies. Also, the findings from previous studies showed that the response to L-cone-directed stimuli produced a stronger response than to the M-cone-directed stimuli. Our results showed an equal or a slightly stronger M-cone response amplitude in the predominant number of subjects. However, the ratios reported from those studies using multifocal VEPs and central mfERG were close to unity [15, 16, 18]. Langrova and colleagues tried to elicit the cone amplitude ratio dominated at the ganglion cell level using the multifocal pattern ERG and they concluded that the cone ratio decreased in the inner retinal level due to gain normalizations [16]. The current inner retinal data from the IC was also observed to be lower than the DC data, confirming that there could be some gain neutralizations happening due to spatial antagonism at the inner retinal level. Another significant difference that prevails in literature is the kind of stimulator used. CRT monitor was conventionally used for reporting the standard and research protocols of mfERG paradigms in the past. However, due to the advancement of visual display technology in the previous two decades, the LED monitor has become the dominant trend to replace conventional CRTs. To the best of our knowledge, this is the first study conducted to test the utility of LED monitors in silent substitution-based ERG study. It is important to be mindful of the colour presenting characteristics of the LED monitor, which are different from those of CRT, and could potentially be a reason for the occurrence of a different cone ratio from mfERG in the present study.

Compared to the existing literature, the inter-individual variations in L/M cone ratio were also lower in the current study. This was aligned with the properties of cone ratios when red-green stimulus with low temporal flickering frequency (around 8–12 Hz) has been used [31]. Jacob and co-workers, using full-field flicker ERG, showed the influence of the stimulus size on the cone ratio and it was observed that a small macular stimulus showed a L/M-cone ratio close to unity. In the present MOFO setting with four video frames in each MOFO frame, the temporal frequency of the stimulation is around 15 Hz, the L/M cone ratio is close to unity (approx 0.80) in the current study, possibly aligning with the existing literature. It was reported that a cone ratio close to unity and with low inter-individual variation may reflect the red-green opponent channel [9]. Kremers et al. had recommended the addition of spatial variation to flicker ERGs to study the spatial properties of the chromatic and luminance pathway with the influence of retinal eccentricity [32]. This triggers an interesting question: What is the potential of mfERG with the silent substitution method to study the chromatic and luminance activities of the post-receptor pathways?

Another noticeable feature is the influence of various ethnicities on the L- to M-cone ratio data. The current study is focused in the Chinese population, while the previous studies indicated in Fig. 7 were mostly on Caucasians. Kuchenbecker recruited 138 African Americans, 362 Caucasians, 19 Chinese, 17 Koreans and 18 Native Americans and compared their L/M cone ratio through opsin gene sequencing and concluded that the Caucasians had a higher mean L/M cone ratio of around 1.9 compared to other ethnicities. The mean ratio reported in their study for other ethnicities ranged from 0.8 to 1.3 which corresponded with the present study [43]. The current data presented here could be a valuable addition to the existing body of knowledge in this emerging field, where distinct data are crucial for advancing future research. Given the potential opportunities of the silent substitution MOFO mfERG protocol, future work will focus on measuring the L- and M-cone-directed MOFO mfERG paradigms in vision disorders such as refractive errors, macular degeneration and glaucoma, providing insights into cone photoreceptor functions. Additionally, this approach can extend to investigate S-cone responses, with ongoing studies contributing to a deeper understanding of these mechanisms. The cellular origins of DC and IC of achromatic MOFO mfERG confirmed using the pharmacological dissection method [7] this can be applied to L- and M- cone specific ERGs to understand the robustness of the developed protocols.

A significant limitation of this study is the use of commercially available LED monitor and LED light used for the main experiment and bright light adaptation experiment respectively. This somewhat limits the stimuli manipulation and restricts the testing conditions. The use of four-primary stimulator to achieve triple silent substitution can be advantageous to eliminate intrusions from non-targeted photoreceptor types. However, the three-primary LED stimulator used in the present study, makes slight intrusions from undesirable photoreceptors unavoidable. Additionally, the absence of dichromat data for validation poses another limitation. The cone fundamentals were not individually calibrated for each participant. Instead, standardised Stockman and Sharpe fundamentals for a CIE standard observer were applied. This may introduce inter-subject variability in the effective cone contrasts due to individual differences in pre-retinal filtering and macular or lens pigment density. Also, measuring individual differences in retinal-level colour vision is inherently challenging, as most colour vision tests rely on subjective psychophysical responses involving cognitive processes. Our study circumvents this limitation by employing an objective electrophysiological approach (electroretinography), providing direct assessment of cone photoreceptor function independent of higher-order visual processing. Regarding the bright light adaptation, future work preferably should incorporate colour vision testing (e.g., Ishihara plates, anomaloscope matching, or cone-directed psychophysical tasks) to directly verify that the protocol achieves the intended cone selectivity. This would refine and strengthen the technical validation of the approach.

In conclusion, the mean L/M cone amplitude ratio based on the MOFO mfERG with the silent substitution method was found to be 0.84 for DC and 0.77 for the IC, with a small inter-individual variation for the Chinese population. Different characteristics of DC and IC related to L- and M-cone were demonstrated. The equal response from L- and M-cone photoreceptors in the present data could possibly reflect the activities of the chromatic channel. The incorporation of silent substitution in global flash mfERG presentation may explore a new direction in studying photoreceptor physiology in terms of clinical and research aspects.

## Data Availability

Data available on request from the authors.

## Appendix 1:

The appendix presents the computation of the coefficient matrix necessary for achieving a cone-directed stimulus. When the total quantal catch produced by the three primaries in each of the 3 cone types equals the quantal catch produced by the spectral test light, the match is said to have been achieved.

$$\left( {\begin{array}{*{20}c} L \\ M \\ S \\ \end{array} } \right) = \left( {\begin{array}{*{20}l} {LR} \hfill & {LG} \hfill & {LB} \hfill \\ {MR} \hfill & {MG} \hfill & {MB} \hfill \\ {SR} \hfill & {SG} \hfill & {SB} \hfill \\ \end{array} } \right)\times\left( {\begin{array}{*{20}c} r \\ g \\ b \\ \end{array} } \right)$$

Where; *r(L*_*R*_ + *M*_*R*_ + *S*_*R*_*)* is the effective quantal catch produced by r units of R.

Representation of Effective Quantal catch produced in individual cone types:

$$\left( {rL_{R} + gL_{G} + \, bL_{B} } \right) \, + \, \left( {rM_{R} + gM_{G} + \, bM_{B} } \right) \, + \, \left( {rS_{R} + gS_{G} + \, bS_{B} } \right) \, = \, rR \, + \, gG \, + \, bB \, = \, U$$

Where; *(rL*_*R*_ + *gL*_*G*_ + *bL*_*B*_*)* is the effective quantal catch produced in L cone by test light U.

The above linear relationship can be expressed in terms of matrix notation.

$$\left( {\begin{array}{*{20}c} L \\ M \\ S \\ \end{array} } \right) = \left( {\begin{array}{*{20}c} {LR} & {LG} & {LB} \\ {MR} & {MG} & {MB} \\ {SR} & {SG} & {SB} \\ \end{array} } \right)\times\left( {\begin{array}{*{20}c} r \\ g \\ b \\ \end{array} } \right)$$

This matrix can be represented in the form of an equation as;

$$LMS \, = \, LMS\_rgb \, \times \, rgb$$

This equation can also be used to obtain rgb using the inverse matrix of LMS_rgb as follows.

$$rgb \, = \, rgb\_LMS \, \times \, LMS$$

Since the human eye has three cones and three primaries, there are 9 components in the *rgb_LMS* matrix representing the effective quantal catches. For instance, the effective quantal catch produced in L cone by green LED can be represented as;

$$L_{G} = \, \smallint \, L\left( \lambda \right) \, G\left( \lambda \right) \, d\lambda$$

In this context, L(λ) represents the L cone fundamentals, while G(λ) denotes the emission spectrum of green light. The matrix coefficients can be calculated using the formula mentioned above along with the constant k. The value of k varies for each cone type—kL, kM, kS—depending on τλmax, which is the product of ocular media transmissivity and the absolute absorption coefficients at the wavelength where each cone has maximum absorption probability. The constant k itself is calculated as the product of the foveal cone collecting area and τλmax, divided by the square of the distance between the nodal point of the lens and the retina [44, 45]

$$\left( {\begin{array}{*{20}c} L \\ M \\ S \\ \end{array} } \right) = \left( {\begin{array}{*{20}l} {180561} \hfill & {274969} \hfill & {18698} \hfill \\ {59702} \hfill & {240706} \hfill & {21293} \hfill \\ {471} \hfill & {1685} \hfill & {13844} \hfill \\ \end{array} } \right)\times\left( {\begin{array}{*{20}c} r \\ g \\ b \\ \end{array} } \right)$$

ove, is the coefficient matrix for our LED emissions. Each component of the matrix means the number of quanta absorbed in a single cone in one second when the full intensity of the corresponding light is used. This coefficient matrix can also be used to obtain the quantal catches corresponding to different intensities of the *R, G, B* lights. We achieved *L* cone modulation by altering equation (a) as follows.

For instance, for achieving a maximal change in the quantal catches of L-cones, ∆L is set to ∆L max while ∆M and ∆S are set to 0.

For the same condition, red light is set at maximum intensity that is ∆r = 1.

$$\left( {\begin{array}{*{20}c} {1.0} \\ {\Delta g} \\ {\Delta b} \\ \end{array} } \right) = rgb\_LMS \times \left( {\begin{array}{*{20}c} {\Delta L\max } \\ 0 \\ 0 \\ \end{array} } \right)$$

Solving the above matrix relation, we obtained.

$$\Delta L \, \max \, = 112383.41,\Delta g = 0.25 and\Delta b = 0.0039$$

Using the *∆r, ∆g* and *∆b* values, the respective luminance for the L-cone-directed condition and the two states for the multifocal frames were set. Since the input–output luminance relation slightly deviated from linearity, a look-up Table (1 cd/m2 step) was used to set the required luminance.
